# A shared responsibility for the future of prevention – Aligning ASPC and AJPC

**DOI:** 10.1016/j.ajpc.2026.101439

**Published:** 2026-02-23

**Authors:** Michael D. Shapiro

**Affiliations:** Cardiology and Molecular Medicine, Center for Prevention of Cardiovascular Disease, Department of Cardiovascular Medicine, Wake Forest University School Medicine, Winston-Salem, NC 27157, United States

When I talk with colleagues, I hear the same frustrations. Despite major advances, the gap between knowledge and patient care remains wide. We generate high-quality evidence, publish it, debate it, and update guidelines, but translation into real-world care is slow and often incomplete.

Most of us practice in environments where time is limited, systems are fragmented, and incentives are often misaligned with long-term risk reduction. Risk assessment tools are inconsistently applied. Proven therapies are underutilized. A person's access to care depends more on where they are seen than on what the evidence supports. We know prevention strategies are effective. But we need to build the structures and pathways needed to make it work for most patients, not just those fortunate enough to receive care in well-resourced systems. The gap between evidence and practice is the central challenge of our field. Closing it will require a shift in what we value and elevate. Implementation, care delivery, and education need to be prioritized. They are the mechanisms through which evidence becomes care.

This moment also coincides with a transition in leadership at the American Journal of Preventive Cardiology, with Dr. Khurram Nasir assuming the role of Editor-in-Chief. His work has consistently emphasized risk assessment, population health, and real-world impact, areas that sit at the core of modern prevention. Under his leadership, the alignment between ASPC and AJPC creates an opportunity to more tightly connect clinical questions arising from practice with rigorous scholarship that can shape care at scale. This will also provide direction for the field.

One group that stands to benefit most from this alignment is fellows and early-career clinicians. Training in preventive cardiology today is highly variable. Exposure, expectations, and mentorship differ widely across programs and institutions. A closer alignment between ASPC and AJPC helps bring greater clarity to what we value and what we expect, both in practice and in scholarship. It helps set clearer standards for competencies while also offering structure and encouragement for those early in their careers. By elevating real-world research, implementation studies, and multidisciplinary care through a respected journal, this partnership helps fellows see how their work can translate into real impact, academic recognition, and durable careers in prevention.

Ultimately, prevention succeeds only when knowledge reaches patients in meaningful ways. Aligning our society and our journal around that shared goal carries responsibility, because it shapes what we prioritize and what we choose to act on. It reflects a clear commitment to narrowing the gap between what we know and what we do, and to building a field that lives up to its promise. ASPC and AJPC are aligned in purpose, priorities, and responsibility.

Michael D. Shapiro

President, American Society for Preventive Cardiology

## CRediT authorship contribution statement

**Michael D. Shapiro:** Conceptualization, Writing – original draft, Writing – review & editing.

## Declaration of competing interest

The authors declare the following financial interests/personal relationships which may be considered as potential competing interests:

Michael D. Shapiro reports a relationship with Amgen, Arrowhead, Ionis, Novartis, New Amsterdam, Merck, Regeneron, Aidoc, Novo Nordisk, and Tourmaline that includes: consulting or advisory. Michael D. Shapiro reports a relationship with Amgen, Arrowhead, Boehringer Ingelheim, Esperion, Novartis, Ionis, Merck, New Amsterdam, Lilly, and Cleerly that includes: funding grants. If there are other authors, they declare that they have no known competing financial interests or personal relationships that could have appeared to influence the work reported in this paper.

